# Impact of violated high‐dose refuge assumptions on evolution of *Bt* resistance

**DOI:** 10.1111/eva.12355

**Published:** 2016-02-27

**Authors:** Pascal Campagne, Peter E. Smouse, Rémy Pasquet, Jean‐François Silvain, Bruno Le Ru, Johnnie Van den Berg

**Affiliations:** ^1^ Laboratoire Évolution, Génome et SpéciationCNRS UPR9034Unité de Recherche IRD 072Gif‐sur‐YvetteFrance; ^2^ Université Paris‐Sud 11OrsayFrance; ^3^ Department of Ecology, Evolution & Natural ResourcesSchool of Environmental & Biological SciencesRutgers UniversityNew BrunswickNJUSA; ^4^ Noctuid Stem Borers Biodiversity in Africa ProjectEnvironmental Health DivisionInternational Centre for Insect Physiology & EcologyNairobiKenya; ^5^ Institute of Integrative BiologyUniversity of LiverpoolLiverpoolUK; ^6^ School of Biological Sciences ‐ ZoologyNorth‐West UniversityPotchefstroomSouth Africa

**Keywords:** fitness cost, high‐dose, incomplete resistance, insecticide resistance, nonrandom mating, partial dominance, refuge strategy

## Abstract

Transgenic crops expressing *Bacillus thuringiensis* (*Bt*) toxins have been widely and successfully deployed for the control of target pests, while allowing a substantial reduction in insecticide use. The evolution of resistance (a heritable decrease in susceptibility to *Bt* toxins) can pose a threat to sustained control of target pests, but a high‐dose refuge (HDR) management strategy has been key to delaying countervailing evolution of *Bt* resistance. The HDR strategy relies on the mating frequency between susceptible and resistant individuals, so either partial dominance of resistant alleles or nonrandom mating in the pest population itself could elevate the pace of resistance evolution. Using classic Wright‐Fisher genetic models, we investigated the impact of deviations from standard refuge model assumptions on resistance evolution in the pest populations. We show that when *Bt* selection is strong, even deviations from random mating and/or strictly recessive resistance that are below the threshold of detection can yield dramatic increases in the pace of resistance evolution. Resistance evolution is hastened whenever the order of magnitude of model violations exceeds the initial frequency of resistant alleles. We also show that the existence of a fitness cost for resistant individuals on the refuge crop cannot easily overcome the effect of violated HDR assumptions. We propose a parametrically explicit framework that enables both comparison of various field situations and model inference. Using this model, we propose novel empiric estimators of the pace of resistance evolution (and time to loss of control), whose simple calculation relies on the observed change in resistance allele frequency.

## Introduction

Genetically modified crops, expressing insecticidal toxins of *Bacillus thuringiensis* (*Bt*), were first introduced in 1995 and have now been adopted worldwide; by 2010, they had been planted on ~66 Mha of agricultural crop land (James 2011). While *Bt*‐expressing crops have met with considerable success, resistance can arise whenever a pest population develops a genetically based decrease in susceptibility to the toxin (Tabashnik et al. 2009), which may lead in turn to drastic loss of *Bt* crop efficacy under field conditions (i.e., effective field resistance). While resistant mutations have been reported in many cases (Tabashnik et al. 2013), almost two decades after *Bt* crops were first deployed, clearly documented cases of effective field resistance have arisen in only four pests: *Busseola fusca* (South Africa, Van Rensburg 2007), *Spodoptera frugiperda* (Puerto Rico, Storer et al. 2010), *Pectinophora gossypiella* (India, Dhurua and Gujar 2011), and *Diabrotica virgifera virgifera* (USA, Gassmann et al. 2011).

Much attention has been devoted to the pace of resistance evolution (Tabashnik et al. 2013), as well as to developing operational strategies that can delay (Alstad and Andow 1995) or eventually reverse it (Carrière et al. 2010). Among them, the high‐dose/refuge (henceforth, HDR) strategy, resulting in a lowered selection pressure on susceptible individuals (Carrière et al. 2010), has generally been effective (Huang et al. 2011), particularly in the USA, where its proper implementation has seldom led to loss of control (Tabashnik et al. 2013). This strategy amounts to planting nonresistant cultivars within or surrounding *Bt*‐crop plantings, allowing the survival of some susceptible individuals in a *Bt*‐dominated environment. If susceptible alleles (S) in the pest are dominant and rare resistant mutants (R) are completely recessive, then rare resistant individuals (RR) emerging from *Bt* plants will mate preferentially with susceptible individuals (SS) emerging from refuge plants. Crosses between (RR) and (SS) parents yield (RS) progeny, so if the dose of *Bt* toxin expressed is high enough to kill 100% of heterozygous (RS) larvae, the HDR strategy should strongly delay evolution of pest resistance to *Bt* toxins. Recommended refuge fractions for *Bt* crops have ranged from ~5% to 50% of crop acreage in the USA (Bates et al. 2005), depending notably on whether or not they were also sprayed with insecticide.

Theory shows that optimal efficiency of the HDR strategy is guaranteed when: (i) the genetic bases of resistance in natural populations and the dose of toxin expressed by the plant result in functionally recessive expression in the pest; (ii) mating is random among pest genotypes, with regard to *Bt* resistance; and (iii) the frequency of resistant mutants is low. The available data suggest that low background frequencies (*q*_0_) of resistance alleles are associated with sustained susceptibility to *Bt* toxin (Tabashnik et al. 2013), so most modeling studies have explored cases where (*q*_0_ ≤ 0.001) (e.g., Tyutyunov et al. 2008).

Success of the HDR strategy depends on the dominance level of the resistance allele (1 > *h *>* *0), with *h *=* *0 corresponding to a recessive trait and *h *=* *1 to a dominant trait (Wright 1934). It also depends on the rate of nonrandom mating for resistant genotypes (*F *>* *0), resulting in excesses of resistant homozygotes (RR), relative to panmictic expectation. Success also depends on the background frequency of (or rate of mutation to) resistant alleles (*q*_0_ > 0), as well as to the proportion (1 − *ω*) of the susceptible (refuge) crop that is planted.

The fraction of *Bt* crop planted in the landscape (*ω*) is expected to scale with the proportion of susceptible pest individuals killed by the toxin. A lack of refuge planting in India and China has apparently allowed rapid evolution of *P. gossypiella* resistance to Cry1Ac *Bt* cotton (Tabashnik et al. 2013). Similarly, low compliance among South African farmers in planting the recommended fraction of refuge *Z. mays* crop might have hastened the evolution of *Bt* resistance in the stem borer (*B. fusca*) (Kruger et al. 2012).

A review of documented cases of field monitoring has shown that rapid evolution of resistance occurs predominantly when the initial frequency of resistance allele (*q*_0_) was above the threshold of detectability (Tabashnik et al. 2013). It has also been shown, however, that sustained susceptibility to *Bt* toxins can be achieved in the field, even when (*q*_0_ > 0.001), when coupled with a high fraction (1 − *ω*) > 40% of refuge acreage (Tabashnik et al. 2013).

Either failure to achieve a high‐dose concentration of toxin in plant tissues and/or the presence of partially dominant (*h > *0) resistance alleles yields a surviving fraction of heterozygous (RS) larvae on *Bt* plants, which compromises HDR success. Notwithstanding the potential problems, recessive inheritance has been supported by numerous studies of both laboratory‐selected and field‐evolved *Bt* resistance (e.g., Ferré and Van Rie 2002; Tabashnik et al. 2003). On the other hand, it is notoriously difficult to estimate dominance (*h*) levels reliably, under either field conditions (Moar et al. 2008; Tabashnik et al. 2008) or in the laboratory, largely attributable to concentration‐dependent effects of the toxin (Gould et al. 1995; Tabashnik et al. 2002). There have also been more striking cases, for which (strong) partially dominant (*h *>* *0.5) resistance has been observed, probably stemming from diverse inheritance or biochemical bases of resistance in a variety of different organisms (Zhang et al. 2012; Campagne et al. 2013; Jin et al. 2013).

Likewise, any elevated tendency (*F *>* *0) for resistant individuals (emerging from the *Bt* crop) to mate with each other, rather than with susceptible individuals (emerging from the refuge crop), profoundly increasing the frequency of resistant (RR) homozygotes among the progeny [fr(RR progeny) = (*q* ^2^ + *Fpq*)] and compromising the efficacy of the HDR strategy. Promoting mating between resistant and susceptible individuals depends on both, the spatial structure of the *Bt* crop and refuge blocks and individual postemergence dispersal patterns (Alstad and Andow 1995). Many pest populations conform satisfactorily to Hardy–Weinberg expectations for selectively neutral markers (Han and Caprio 2002; Endersby et al. 2007; Krumm et al. 2008; Kim et al. 2009), suggesting a mating regime close to random, but whether that same condition obtains for genetic markers under strong and spatially structured *Bt* selection remains unclear. In spite of extensive genetic mixing and low inbreeding levels in the moth *Ostrinia nubilalis* (Bourguet et al. 2000), Dalecky et al. (2006) have demonstrated that this species would be prone to positive assortative mating in *Bt*‐crop context. Indeed, mating between resistant individuals originating from a single *Bt* planting could reach a few percent, as a consequence of limited premating dispersal. The effects of the spatial structure of refuge plantings have been both contentious and extensive (Onstad et al. 2011). Some modeling studies have suggested that large block refuges could be more efficient in delaying resistance evolution than scattered refuges (Tyutyunov et al. 2008); others have suggested that seed blends (yielding a spatial mixture of *Bt* and non‐*Bt* plants in the field) could provide at least as much HDR durability as block refuges (Pan et al. 2011). In practice, we still know very little about the empiric rates of nonrandom mating under field conditions for most pests.

Other crucial factors that might delay the evolution of resistance have been assessed in different pest species (Gassmann et al. 2009), among them: incomplete resistance, fitness cost, and the dominance of the fitness cost. Incomplete resistance denotes situations where the fitness of resistant individuals on *Bt* plants (*V*_RR_) is lower than the fitness of susceptible individuals on non‐*Bt* plants (*U*_SS_), i.e., when (*V*_RR_ < *U*_SS_), which reduces the selective advantage of resistant individuals in mixed plantings of *Bt* and non‐*Bt* plants (Carrière et al. 2006). Fitness cost arises when a resistance allele reduces the fitness of homozygotes (RR) in environments that are toxin‐free, so that (*U*_SS_ − *U*_RR_ > 0) (Tabashnik et al. 2014). Fitness cost may also exhibit a range of dominance levels (0 ≤ *g *= (*U*_SS_ − *U*_RS_)/(*U*_SS_ − *U*_RR_) ≤ 1) as shown in Table 1. Available data suggest that a recessive (*g *≈* *0) fitness cost of 25% (*U*_SS_ = 1, *U*_RR_ = 0.75, *U*_SS_ − *U*_RR_ = 0.25) might be a reasonable average (Gassmann et al. 2009). Management accounting for fitness cost may strengthen the effects of the HDR strategy in delaying the evolution of resistance (e.g., Higginson et al. 2005).

**Table 1:** Summary of allelic fitness values, under the different parametric assumptions of the model

Model parameters	SS	RS	RR	Planting fraction
Frequencies	*p* ^2^ * + pqF*	2*pq*(1 − *F*)	*q* ^2^ * + pqF*	
*Bt* fitness	*V*_SS_ < *V*_RR_	*V*_RS_ = *V*_SS_ * + h*(*V*_RR_ − *V*_SS_)	*V*_RR_	*ω*
Refuge fitness	*U*_SS_	*U*_RS_ = *U*_RR_ * + g*(*U*_SS_ − *U*_RR_)	*U*_SS_ > *U*_RR_	(1 − *ω*)
Average allelic fitness values – *Bt* crop				
	${\tilde{V}}_{S}=\left[\left(p+qF\right)\cdot V_{\mathrm{SS}}+q\cdot \left(1-F\right)\cdot V_{\mathrm{RS}}\right]$		${\tilde{V}}_{R}=\left[\left(q+pF\right)\cdot V_{\mathrm{RR}}+p\cdot \left(1-F\right)\cdot V_{\mathrm{RS}}\right]$	
Average allelic fitness values – refuge crop				
	${\tilde{U}}_{S}=\left[\left(p+qF\right)\cdot U_{\mathrm{SS}}+q\cdot \left(1-F\right)\cdot U_{\mathrm{RS}}\right]$		${\tilde{U}}_{R}=\left[\left(q+pF\right)\cdot U_{\mathrm{RR}}+p\cdot \left(1-F\right)\cdot U_{\mathrm{RS}}\right]$	
Weighted average allelic fitness values – both crops				
	${\tilde{W}}_{S}=\left[\left(1-\omega \right)\cdot {\tilde{U}}_{S}+\omega \cdot {\tilde{V}}_{S}\right]$		${\tilde{W}}_{R}=\left[\left(1-\omega \right)\cdot {\tilde{U}}_{R}+\omega \cdot {\tilde{V}}_{R}\right]$	

Failure of standard HDR assumptions (Huang et al. 2011; Tabashnik et al. 2013) has led to occasional resistance development (Tabashnik et al. 2014), and the matter needs further exploration, both theoretically and empirically. Using Wright's (1942) classical genetic model, we here explore the sensitivity of resistance evolution to assumptions of strict randomness in mating and strictly recessive resistance alleles. This study is aimed at: (i) testing the robustness of the model when *F* and/or *h* might be slightly higher than 0; (ii) assessing the extent to which nonrecessive expression and nonrandom mating may balance the effects of fitness cost (*U*_SS_ − *U*_RR_ > 0 and *g *>* *0) and incomplete resistance (*U*_SS_ − *V*_RR_ > 0); (iii) evaluating whether violations of model assumptions impact the expected time elapsed before buildup of resistance in the pest threatens the efficacy of the *Bt* crop itself.

## Modeling evolution of *Bt* resistance

Resistance is considered to involve a single locus, with a susceptible allele (S), of frequency *p*, and a resistance allele (R), of frequency *q*. The survival probability of the genotypes RR, RS, and SS is denoted by (*U*_RR_, *U*_RS_, and *U*_SS_) on refuge plants and (*V*_RR_, *V*_RS_, and *V*_SS_) on *Bt* plants (Table 1). The proportion of *Bt* crop in the landscape (*ω*) determines the relative fitness of the three genotypes; for modeling purposes, the spatial distribution of *Bt* and non‐*Bt* plants is considered continuous and random. The net relative fitness values of the three genotypes, emerging from a spatially randomized blend of *Bt* (*ω*) and refuge (1 − *ω*) plants are as follows:

(1) $$\begin{array}{cc} W_{\mathrm{SS}} & =\left(1-\omega \right)\cdot U_{\mathrm{SS}}+\omega \cdot V_{\mathrm{SS}}\\ W_{\mathrm{RS}} & =\left(1-\omega \right)\cdot U_{\mathrm{RS}}+\omega \cdot V_{\mathrm{RS}}\\ W_{\mathrm{RR}} & =\left(1-\omega \right)\cdot U_{\mathrm{RR}}+\omega \cdot V_{\mathrm{RR}} \end{array}$$

Any tendency for preferential mating (to type), whether due to genetically programmed behavioral or spatially imposed dispersal patterns (to or from the refuge crop), will result in assortative mating (*F* > 0) among newly emerging individuals. Given these genotypic fitness values, the parental genotypic frequencies, and the value of (*F*) (Table 1), we define the average relative allelic fitness values on the refuge crop as:

(2a) $$\begin{array}{cc} {\tilde{U}}_{S} & =[\left(p+qF\right)\cdot U_{\mathrm{SS}}+q\cdot \left(1-F\right)\cdot U_{\mathrm{RS}}]\\ {\tilde{U}}_{R} & =[\left(q+pF\right)\cdot U_{\mathrm{RR}}+p\cdot \left(1-F\right)\cdot U_{\mathrm{RS}}] \end{array}$$

and on the *Bt* crop as:

(2b) $$\begin{array}{cc} {\tilde{V}}_{S} & =[\left(p+qF\right)\cdot V_{\mathrm{SS}}+q\cdot \left(1-F\right)\cdot V_{\mathrm{RS}}]\\ {\tilde{V}}_{R} & =[\left(q+pF\right)\cdot V_{\mathrm{RR}}+p\cdot \left(1-F\right)\cdot V_{\mathrm{RS}}] \end{array}$$

At landscape level, we can then define (see Table 1 and Appendix S1) weighted average allelic fitness values (${\tilde{W}}_{R}$ and ${\tilde{W}}_{S}$ for the collective population (Table 1):

(3) $$\begin{array}{cc} {\tilde{W}}_{S}=\left[\left(1-\omega \right)\cdot {\tilde{U}}_{S}+\omega \cdot {\tilde{V}}_{S}\right]\;\;\text{and}\;\;\\ & {\tilde{W}}_{R}=\left[\left(1-\omega \right)\cdot {\tilde{U}}_{R}+\omega \cdot {\tilde{V}}_{R}\right] \end{array}$$

Standard theory (Wright 1942) shows that the change in the frequency of the (R) allele over a single discrete generation depends on the average fitness of the advantageous allele over the population average:

(4) $$\begin{array}{cc} \Delta q=\left(q^{\prime }-q\right)=q\cdot \left[\frac{{\tilde{W}}_{R}}{\bar{W}}-1\right],\;\\ & \text{where}\;\bar{W}=\left[q\cdot {\tilde{W}}_{R}+p\cdot {\tilde{W}}_{S}\right] \end{array}$$

It is convenient to define an equivalent form, using *y *= *q*/(1 − *q*) = (*q*/*p*), so that (4) can be replaced with a more convenient analogue:

(5) $$\begin{array}{cc} \Delta y=\left[\frac{{\tilde{W}}_{R}}{{\tilde{W}}_{S}}-1\right]\cdot y\\ & =\left[\frac{\omega \cdot {\tilde{V}}_{R}+\left(1-\omega \right)\cdot {\tilde{U}}_{R}}{\omega \cdot {\tilde{V}}_{S}+\left(1-\omega \right)\cdot {\tilde{U}}_{S}}-1\right]\cdot y=\tilde{\Lambda }\cdot y \end{array}$$

where $\tilde{\Lambda }$ accounts for all the parameters in the model, in its most general form (Table 1). In practice, *q* may either increase (Δ*y* > 0, when ${\tilde{W}}_{R}$ > ${\tilde{W}}_{S}$) or decrease (Δ*y* < 0, when ${\tilde{W}}_{R}$ < ${\tilde{W}}_{S}$), while the sets of parameters for which Δ*y* = 0 (0 <  *q* < 1) delineate two alternative trajectories of the resistance allele frequency. Equation (5) expresses a balance between the selective advantage of susceptible individuals on refuge and that of resistant individuals on *Bt* crop, balanced against the refuge crop fraction (1 − *ω*). Comparing the values of ${\tilde{W}}_{R}$ and ${\tilde{W}}_{S}$ amounts to comparing $\left({\tilde{U}}_{S}-{\tilde{U}}_{R}\right)/\left({\tilde{V}}_{R}-{\tilde{V}}_{S}\right)$ with *ω*/(1 − *ω*). If $\left({\tilde{U}}_{S}-{\tilde{U}}_{R}\right)/\left({\tilde{V}}_{R}-{\tilde{V}}_{S}\right)$ > *ω*/(1 − *ω*), the resistant allele (R) increases in frequency. Conversely, if $\left({\tilde{U}}_{S}-{\tilde{U}}_{R}\right)/\left({\tilde{V}}_{R}-{\tilde{V}}_{S}\right)$ < *ω*/(1 − *ω*), the resistant allele (R) decreases in frequency. In practice, the fitness of (RR) individuals on refuge plants may be lower than that of (SS) on refuge plants (*U*_SS_ − *U*_RR_ ≥ 0), labeled a ‘fitness cost’ (e.g., Gassmann et al. 2009; Tabashnik et al. 2014). Moreover, heterozygote (RS) fitness on the refuge crop may also show partial dominance, yielding (*U*_SS_ > *U*_RS_ > *U*_RR_) on the refuge crop, counterbalanced by (*V*_RR_ > *V*_RS_ > *V*_SS_) on the *Bt* crop. Finally, we must also consider incomplete resistance, cases where (*U*_SS_ > *V*_RR_).

### Time to loss of containment (passage time)

An adaptive resistance allele (R) will increase in frequency from very low to very high, in classic sigmoidal fashion. A convenient criterion used to assess the evolution of resistance is the number of generations (henceforth ‘passage time’) for which the frequency of the resistant (R) allele is lower than some critical frequency in the population (say, *q*_*k*_ = 0.1), as in Tyutyunov et al. (2008). If we denote the initial frequency of the resistant allele (R) as *q*_0_ and that of the ‘critical’ value as *q*_*k*_, then the passage time (*T* ^*k*^) for the allele frequency to increase from (*q*_0_ → *q*_*k*_) may be obtained by iteration of equation (5). Discrete models do not yield closed form solutions for (*T* ^*k*^), but continuous approximations provide relatively simple (and parametrically explicit) approximations (see Felsenstein 2007). We constructed differential equations based on the difference equations, *δy*/*δt* = ∆*y*, and used their solutions to derive an approximate formula for passage times (Appendix S2) for each of several models.

In the general form of equation (5), the increase in resistance allele frequency for a single generation can be calculated, based on the difference between *y′* and *y*. To solve the differential equation based on the difference equation (5), $\tilde{\Lambda }$ may be easily rewritten as a ratio of two linear functions of *y*, so the passage time (time to loss of containment) may be calculated for the general form of the model (Appendix S2). When *y*_0_ is small, the expression for passage time can be further simplified (Appendix S2), and we achieve a relatively simple approximation of $T_{\Lambda }^{k}$ for the case where *V*_SS_ = 0 and *U*_SS_ = 1 (the reference fitness values). Given initial (*q*_0_) and critical (*q*_*k*_) frequencies of the (R) allele, the approximate passage time can be written (in terms of *y *= *q/p*), as (see Appendix S2 for the full expression):

(6) $$T_{\Lambda }^{k}\approx \frac{1-\omega }{\omega \cdot \varepsilon \cdot V_{\mathrm{RR}}-\left(1-\omega \right)\cdot \chi \cdot \left(1-U_{\mathrm{RR}}\right)}\cdot \text{ln}\;\left(\frac{y_{k}}{y_{0}}\right)$$

where *ε *= (*F *+ *h* − *F·h*) and *χ *= (*F *+ *g* − *F·g*) capture the essence of deviations from classic HDR assumptions on *Bt* and refuge crops, respectively.

**Figure 1:**
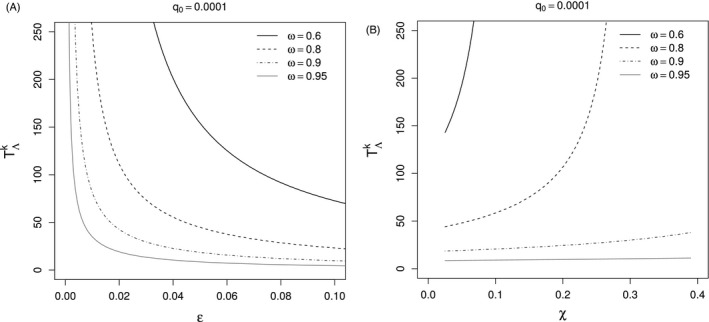
Passage time $T_{\Lambda }^{k}$ (generations) from *q*_0_ = 10^−4^ to *q*_*k*_ = 10^−1^, the critical allele frequency of the resistance allele (R), under two different scenarios. (A) Effects of deviations from the assumption *ε *= 0 (*F *=* *0 = *h*) on passage time $T_{\Lambda }^{k}$ with varied *Bt*‐crop fraction: 0.6 ≤ *ω *≤ 0.95. The parameters of the model were set as follow: *V*_RR_ = 0.75, *U*_RR_ = 0.75, *U*_SS_ = 1, *g *=* *0.05. (B) Combined effects of *F* and *g* (*χ *= *F *+ *g* − *Fg*) on passage time $T_{\Lambda }^{k}$. Parameters of the model: *V*_RR_ = 0.75, *U*_RR_ = 0.5, *U*_SS_ = 1, *F *= *h *=* *0.025 (*ε *≈ 0.05) and 0 < *g *<* *0.375 (0 < *χ *< 0.4).

We observe that mild deviations from HDR assumptions (e.g., *ε *= 0.05) dramatically shorten passage time, even when fitness cost and incomplete resistance are substantial (Fig. 1). Equation (6) suggests that the role of fitness cost, in terms of both (1 − *U*_RR_) and (*g*), as well as incomplete resistance, denoted by (*V*_RR_ < *U*_SS_), may have an impact for large fractions of refuge (1 − *ω*) (Fig. 1). When most of the acreage is planted to the transgenic crop (*ω* is elevated), however, substantial levels of fitness cost and incomplete resistance are required to delay resistance evolution substantially. Transgenic crops will presumably be dominant in the landscape, so the sensitivity of passage time to the deviation parameter *ε *= (*F *+ *h* − *F·h*) is greater than are the protective effects of fitness cost and incomplete resistance. By inflating the frequency of homozygotes (RR), *F* reinforces the role of fitness cost in the second part of the denominator of equation (6), so that the parameter *h* is expected to have a larger effect on $T_{\Lambda }^{k}$ than will *F*, whenever fitness cost and incomplete resistance are sizeable.

**Figure 2:**
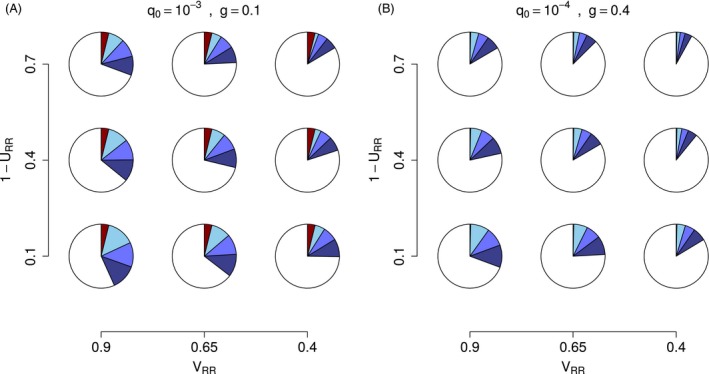
Additional proportion of refuge required (1 − *ω*) to keep passage time $T_{\Lambda }^{k}$ above 40 generations (blue slices) when the model deviates from strict recessivity and strict random‐mating [*ε *= 0, equation (10) red slices]. Two scenarios were envisaged: (A) *q*_0_ = 10^−3^ and *g *=* *0.1, and (B) *q*_0_ = 10^−4^ and *g *=* *0.4. Additional refuge fractions, when deviation increases, were calculated based on equations (5) and (6): light blue slices represent *ε *≈ 0.02 (*F *=* *0.01 = *h*); *ε *≈ 0.05 (*F *=* *0.025 = *h*), middle blue; *ε *≈ 0.10 (*F *=* *0.05 = *h*), darker blue. Various combinations of parameters were used for incomplete resistance (0.4 ≤ *V*_RR_ ≤ 0.9) and fitness cost (0.1 ≤ (1 − *U*_RR_) ≤ 0.7) with *U*_SS_ = 1.

We further explored the extent to which deviations from the idealized HDR assumptions (*ε *= 0) could be compensated for by increasing the fraction of refuge, fitness cost, and incomplete resistance. We can calculate the minimal fraction of refuge required to achieve a passage time greater than a given number of generations, based on equations (5) and (6) (see also Appendix S2). Insisting on a passage time of at least 40 generations, we assessed refuge requirements, based on our generalized model (Fig. 2). Refuge requirement appeared to depend more on *ε* than on any other feature of the model. While the amount of refuge (1 − *ω*) required to ensure that ($T_{\Lambda }^{k}>40$) generations was typically lower than the minimal requirement of 5% (unsprayed) refuge recommended for the classic model (*ε *= 0), a suitable refuge fraction was higher than 40% (*F *=* *0.05 = *h*) when incomplete resistance and fitness cost were moderate (*V*_RR_ = 0.9) and (1 − *U*_RR_ = 0.1), respectively (Fig. 2). A low refuge requirement (1 − *ω *< 0.10) was only appropriate for fairly incomplete resistance and high values of fitness cost, for example, (*q*_0_ = 10^−4^, *g *=* *0.4, *ε *≤ 0.05, (1 − *U*_RR_)  > 0.4, *V*_RR_ < 0.65). In overview, a sustained efficacy of *Bt* crops over a time horizon of 20 years appears attainable for most multivoltine species, but only with large fractions of refuge.

Along the same lines, some robust strategies might even be needed to ensure that (Δ*y* ≤ 0). In this case, the minimum fraction of refuge preventing an increase in resistance allele frequency, i.e. which guarantees $\left(\omega \cdot \varepsilon \cdot V_{\mathrm{RR}}\right)<\left(1-\omega \right)\cdot \chi \cdot \left(1-U_{\mathrm{RR}}\right)$ when (*q*_0_ → 0), would constitute an interesting benchmark [via equation (5)]:

(7) $$\begin{array}{c} \left(1-\Omega \right)=\underset{q\to 0}{\text{lim}}{\left(1-\omega \right)}_{\left[\Delta y=0\right]}=\frac{\varepsilon \cdot V_{\text{RR}}}{\varepsilon \cdot V_{\text{RR}}+\chi \cdot \left(1-U_{\text{RR}}\right)} \end{array}$$

According to equation (7), Δ*y* < 0 may be achieved only if $9\times \left(\varepsilon \cdot V_{\mathrm{RR}}\right)<\chi \cdot \left(1-U_{\mathrm{RR}}\right)$, for a refuge fraction of 10%; or $4\times \left(\varepsilon \cdot V_{\mathrm{RR}}\right)<\chi \cdot \left(1-U_{\mathrm{RR}}\right)$, for a refuge fraction of 20%, which clearly refers to cases where incomplete resistance (*V*_RR_ « 1), fitness cost (1 − *U*_RR_ » 0), and the dominance of this cost (*g* » 0) are considerable. Given that fitness cost might average at (1 − *U*_RR_) ≈ 0.25 and might be a rather recessive trait (*g *<* *0.25), a decrease in resistance allele frequency might not be obtained for (1 − Ω) < 0.3, in most cases.

### Some simpler cases

While the general model illustrates the sensitivity of the pace of resistance evolution to even mild deviations from the ideal HDR assumptions, it is also useful to examine some special cases that elucidate particular features of the general problem, all involving relative fitness of SS pest genotypes (*U*_SS_ = 1) on refuge plants and (*V*_SS_ = 0) on *Bt* plants, where resistance is complete (*V*_RR_ = 1) and where there is no fitness cost (*U*_RR_ = *U*_RS_ = *U*_SS_ = 1).

#### Basic HDR model (h = F = 0)

In a strictly recessive model, the (RR) individuals are resistant to *Bt* (*V*_RR_ = 1), but both RS and SS individuals are fully susceptible (*V*_SS_ = 0, and *V*_RS_ = *h *=* *0). Mating is assumed to be random, with respect to the genetic locus in question (*F *=* *0). The proportion of *Bt* crop in the landscape (*ω*) alone determines the relative fitness of the three genotypes. Under such conditions, $\tilde{\Lambda }$ in equation (5) simplifies to:

(8) $$\tilde{\Lambda }=\tilde{A}=\left(\frac{q\cdot \omega }{1-\omega }\right)$$

The rate of resistance increase is determined by the ratio of (*Bt*/Refuge) crop fractions, [*ω*/(1 − *ω*)]. If *y* is initially low, the inflation due to the ratio ($\tilde{A}$) is moderate if the refuge fraction is above 10% [i.e., as long as (*ω *< 0.9)]. There is very slow increase in the frequency of the (R) allele, at least until (*q* ^2^ > 0.01). We use (7) as the reference frame, against which to gauge the impact of violated HDR assumptions on the rate of resistance evolution.

#### Nonrandom mating (F > 0) and nonrecessive (h > 0) models

Next, we consider both the case of nonrecessive resistance and nonrandom mating, due to mating of relatives or to ‘mating to type’. Nonrandom mating (*F* > 0) elevates the frequency of rare resistant homozygotes (RR), while *h* > 0 increases the fraction of heterozygotes (RS) surviving on *Bt* plants. Either nonrecessive resistance or nonrandom mating results in a dramatic increase in the rate of increase by the (R) allele, and a model with both yields an even more elevated rate of increase (see Appendix S1):

(9a) $$\begin{array}{cc} \Delta y_{\varepsilon >0}=\tilde{\Lambda }\cdot y=\tilde{D}\cdot y,\\ & \text{where}\;\tilde{D}\approx \left[1+\left(\frac{p}{q}\right)\cdot \left(F+h-F\cdot h\right)\right]\cdot \tilde{A} \end{array}$$

with

(9b) $$\frac{\Delta y_{\varepsilon >0}}{\Delta y_{\varepsilon =0}}=\left(\frac{\tilde{D}}{\tilde{A}}\right)\approx \left[1+\left(\frac{p}{q}\right)\cdot \left(F+h-F\cdot h\right)\right]\gg 1$$

The rate of *Bt* resistance evolution is profoundly elevated whenever either *F* and/or *h* » *q*. If both assumptions fail, the effect on the pace of *Bt* resistance evolution is almost additive. As a consequence, the passage time expressions obtained for these two cases present striking differences. Solving the differential equation, for the basic HDR (*ε *= 0) case, yields:

(10) $$T_{\tilde{A}}^{k}\equiv T_{\varepsilon =0}^{k}\approx \left(\frac{1-\omega }{\omega }\right)\cdot \left[\frac{1}{y_{0}}-\frac{1}{y_{k}}+\text{ln}\;\left(\frac{y_{k}}{y_{0}}\right)\right],$$

Equation (10) typically yields long passage times, provided that (*y*_0_ < 0.01). By contrast, for cases where (*h *>* *0) and/or (*F *>* *0), the (1/*y*_0_) term disappears from the passage time equation, and:

(11a) $$T_{\tilde{D}}^{k}\equiv T_{\varepsilon >0}^{k}\approx \left(\frac{1-\omega }{\omega }\right)\cdot \frac{1}{\varepsilon }\cdot \left[\left(\varepsilon -1\right)\cdot \text{ln}\;\left(\frac{\varepsilon +y_{k}}{\varepsilon +y_{0}}\right)+\text{ln}\;\left(\frac{y_{k}}{y_{0}}\right)\right]+\alpha ,$$

where

(11b) $$\alpha =\frac{\varepsilon^{2}+\varepsilon -h}{\varepsilon }\cdot \text{ln}\;\left(\frac{\varepsilon +y_{k}}{\varepsilon +y_{0}}\right)$$

with (*α *< 0.10), provided (*ε* and *q*_*k*_) < (0.1), reducing (equation (11a), (11b)), relative to (10).

The shortening of passage time $\left[T_{\tilde{D}}^{k}-T_{\tilde{A}}^{k}\right]$ depends primarily on the product of [(1 − *ω*)/*ω*] and (*p*_0_/*q*_0_) = (*y*_0_)^−1^_,_ and is dramatic when (*ε *> 0). For any value of *ε* and any starting value of *y*_0_, reducing the refuge fraction (1 − *ω*) shortens passage time. As an example, 5% refuge (1 − *ω *= 0.05) shortens the passage time by a factor of five, relative to the rate for 20% refuge (1 − *ω *= 0.20), everything else being equal. Similarly, for any given value of *ω*, the passage time is drastically reduced whenever (*ε* >> *y*_0_). For example, the set of parameters (*F *= *h *=* *0 → *ε *= 0, *ω *= 0.8, *q*_*0*_ = 10^−4^, *q*_*k*_ = 10^−1^) yields a passage time of $T_{\varepsilon =0}^{k}\approx 2500$ generations, which decreases for (*F *= *h *=* *0.025 → *ε* ≈ 0.05, *ω *= 0.8, *q*_0_ = 10^−4^, *q*_*k*_ = 10^−1^) to a value of $T_{\varepsilon \approx 0.05}^{k}\approx 30$ generations (see also Fig. 1A). In view of the fact that many pest species are multivoltine, empirical loss of containment can be anticipated within 10 years. Even low levels of dominance and/or nonrandom mating can compromise current HDR management protocols, even with high refuge fractions (1 − *ω*).

### Determining passage time from the evolutionary trajectory

Based on the approximation of passage time for the generalized model equation (6), we note that the ratio $T_{\Lambda }^{k}/\text{ln}\;\left(y_{k}/y_{0}\right)$ is a logarithmic mean; i.e., a constant that reflects the pace of resistance evolution. Many monitoring surveys of resistance evolution provided data on observed change in resistance allele frequency (*q*_0_ → *q*_*j*_) over an observed time lapse of *T* ^*j*^ generations. Consistent with equation (6), we can use $T^{k*}$ and *ξ* ^*^  as 1st approximations of passage time to *q*_*k*_ and the pace of resistance evolution, respectively:

(12a) $$\xi^{*}\approx \frac{\omega }{1-\omega }\cdot \varepsilon \cdot V_{\mathrm{RR}}-\chi \cdot \left(1-U_{\mathrm{RR}}\right)=\frac{1}{T^{j}}\cdot \ln\;\left(\frac{y_{j}}{y_{0}}\right)$$

equivalently

(12b) $$T^{k}{}^{*}\approx \frac{1}{\xi^{*}}\cdot \ln\;\left(\frac{y_{k}}{y_{0}}\right)$$

Because it relies on an approximation equation (6) of the general model, $T^{k*}$ is an upper‐bound estimate of passage time ($T^{k*}$ > $T_{\Lambda }^{k}$), but for ($T_{\Lambda }^{k}$ < 100), it is a suitable estimate (see Appendix S2); i.e., ($T_{\Lambda }^{k}{}^{*}$ ≈ $T_{\Lambda }^{k}$). The inverted logarithmic mean ξ* defines the pace of resistance evolution; the higher the evolutionary rate, the shorter the passage time. The utility of such empirical estimates is that, while clearly related to equations (5) and (6), their calculation does not require detailed knowledge of the system, seldom understood well enough together to translate into precise values of the parameters (*ω*,* U*_RR_, *V*_RR_, *g*,* h*, and *F*).

Using published data reporting resistance evolution (Table 2), this exercise suggests a passage time (to *q*_*k*_ = 0.1) of about 10–15 years in the four cases for which suitable time‐lapse data were available (i.e., *q*_*j*_ < 0.1, Table 2). These cases are acknowledged as situations where resistant mutations arose but for which control failure had not (yet) been observed (Tabashnik et al. 2013). In spite of some noticeable differences in terms of survey data, similar values of ξ* have been observed in *H. armigera* in China and Australia, suggesting that this same approach may work reasonably well for similar examples of resistance evolution (Table 2).

**Table 2:** Empirical estimates of pace of resistance evolution ξ* and passage time *T* ^k^* (number of generations) from *q*_0_ to *q*_*k*_ = 0.1, using survey data: *q*_0_, the initial frequency of resistance alleles and *q*_*j*_, the allele frequency measured *T* ^*j*^ generations later. Are considered, 11 cases for which field‐evolved resistance or field resistance has been reported (see Tabashnik et al. 2013)

Case summary					Survey data			Projections		
Pest species	*Bt* crop	Toxin	Country	Gener/Year	*q*_0_	*q*_*j*_	*T* ^*j*^	ξ*	*T* ^k^* Gener	Passage time (years)
*Busseola fusca*	Corn	Cry1Ab	South Africa	2	*a*	>0.1	<16	>0.336	NA	NA
*Diatraea saccharalis*	Corn	Cry1Ab	USA	4–5	0.0023	0.018	27	0.076	50.5	11.2
*Helicoverpa armigera*	Cotton	Cry1Ac	China	3–5	0.0058	0.075	36	0.069	40.5	10.5
*Helicoverpa armigera*	Cotton	Cry2Ab	Australia	3–5	0.0033	0.021	28	0.066	52.5	13.1
*Helicoverpa punctigera*	Cotton	Cry2Ab	Australia	3–5	0.0010	0.0091	28	0.093	54.5	13.6
*Helicoverpa zea*	Cotton	Cry1Ac	USA	3	0.0008	>0.1	<18	>0.273	NA	NA
*Helicoverpa zea*	Cotton	Cry2Ab	USA	3	0.0004	>0.1	<12	>0.471	NA	NA
*Ostrina furnacalis*	Corn	Cry1Ab	Philippines	6	*a*	>0.1	36	>0.130	NA	NA
*Pectinophora gossypiella*	Cotton	Cry1Ac	China	3	*a*	>0.1	39	>0.120	NA	NA
*Pectinophora gossypiella*	Cotton	Cry1Ac	India	4–6	*a*	>0.1	<30	>0.156	NA	NA
*Spodoptera frugiperda*	Corn	Cry1F	USA	10	*a*	>0.1	<30	>0.156	NA	NA

*a*, no empirical estimate of *q*_0_ is available; in such cases, *q*_0_ < 0.001 was assumed to provide an estimate of ξ*. NA, cases for which *q *>* *0.1 occurred within *T* ^*j*^, no projections of passage time were performed.

## Discussion

While iterative genetic simulations of resistance evolution have been used to compare theoretical expectations and empirical data (e.g., Tabashnik et al. 2008; Jin et al. 2015), we have here defined parametrically explicit predictions of the rate of evolution. We embedded our analyses in a general model, which should be useful for modeling a variety of single gene responses to selection in diploid pest organisms. Our approach is complementary to simulations of demogenetic and spatially explicit models, which may include additional levels of realism as well as increasing the number of parameters. Our model reveals contrasting outcomes that reflect the stringency of the HDR assumptions. Indeed, in the simplest cases, the structure of passage time equations differ drastically, depending on whether *ε* is assumed to be strictly ‘0’ or not (see equations (10) and 11a).

The equations reveal the parameters of primary importance in the generalized model to be (*q*_0_, *ω*,* ε*, and χ). By lowering the selective pressure on pest populations, the refuge strategy has been widely successful in delaying the evolution of *Bt* resistance in some major pest species since *Bt* crops were first deployed, 15 years ago (Huang et al. 2011). Notwithstanding the ensuing success, Tabashnik et al. (2013) have reported field‐evolved resistance in 13 of 24 examined cases. Equations derived from a Wright‐Fisher model show that passage time depends primarily on the (refuge/*Bt* crop) ratio (1 − *ω*)/*ω*, but also on the counterbalance between the benefits (*ε*) of resistant (R) alleles on *Bt* crops and those of susceptible (S) alleles on refuge crops (χ), highlighted in equation (12a). The utility of incorporating these countervailing adaptive payoffs in particular designs of the refuge strategy has been addressed by a number of studies (see Gassmann et al. 2009; Tabashnik et al. 2009), but wherever crops expressing insecticidal toxins dominate the landscape, the generalized version of the model is much more sensitive to (*ε*) than to (χ). Indeed, the effects of a recessive fitness cost of 25% (*U*_SS_ − *U*_RR_ = 1 − 0.25), which might be a reasonable average across species (Gassmann et al. 2009), appear limited whenever (*ε *> 0.01) and (*ω *> 0.7). Given the sensitivity of the model to low values of *ε*, the question arises of how small deviations from classic HDR assumptions (*ε*) can be empirically detected, especially with respect to that of recessive resistance and random mating.

### Partial dominance

Foremost, the difficulty of accurately estimating degrees of partial dominance under field conditions has been emphasized (Moar et al. 2008; Tabashnik et al. 2008). Although laboratory bioassays are indisputably useful for monitoring resistance evolution, the extent to which the dominance index (*h*), estimated under laboratory conditions, is an accurate indicator of field dominance is unclear (Bourguet et al. 1996). Indeed, both larval susceptibility to *Bt* toxin and the dominance level of any resistance are typically dosage dependent (Gould et al. 1995; Tabashnik et al. 2002). It follows that an estimate of dominance is highly context specific and its accuracy might be well below the standards that reliable predictions would require.

Assessing the partial dominance of R alleles at early stages of resistance evolution remains a challenge, since such alleles are rare, of potentially different mutational origins, and may catalyze divergent biological functions (Zhang et al. 2012; Jin et al. 2013). In addition, seasonal variation in toxin concentration within plant tissues may translate into temporal variation in functional dominance (Carrière et al. 2010). In a recent review study (Tabashnik et al. 2013), none of the 10 cases for which resistance had evolved to the point where more than 1% of individuals had become resistant could be considered ‘high dose’. In addition, there have been a few published cases of newly emerging resistance alleles showing partial dominance under field conditions (Campagne et al. 2013; Jin et al. 2013). We may yet discover that *Bt* strategies based on a strictly recessive resistance assumption are overly vulnerable to the range of empirical evolutionary responses under field conditions.

### Nonrandom mating

Secondly, the amount of nonrandom mating entrained by refuge structure and individual premating movement is not well understood. Generally, estimates of the randomness of mating often lack statistical power. The limited resolution of the genetic markers that have been routinely deployed in pest species (allozymes, AFLPs), or the frequent occurrence of null alleles in co‐dominant genetic markers (microsatellites), has constrained our ability to detect small deviations from panmictic population structure, especially in Lepidopteran pests (Zhang 2004). For many population genetic studies of moth pests, the analytical power has been sufficient to detect only substantial deviations (*F*‐values > 0.1) from Hardy–Weinberg frequencies (e.g., Bourguet et al. 2000; Han and Caprio 2002; Endersby et al. 2007; Kim et al. 2009). As a consequence, low levels of local nonrandom mating, crucial for HDR strategy, could not really be detected in pest species. We have here assumed an unstructured refuge/*Bt*‐crop distribution and therefore dealt with effective fractions of refuge and *Bt* crop. The extent to which planted refuge within a field and landscape refuge (non‐*Bt* farms) translate into comparable fractions of effective refuge is a pest‐specific question that will need further clarification, particularly in terms of empirical data on actual pest species dispersal dynamics. As highlighted by Bourguet et al. (2000), high levels of gene flow within and among populations do not necessarily translate into a random‐mating pattern, either in general or with regard to genotypes at *Bt*‐relevant loci. It is noteworthy that assortative mating regimes may only be evident for loci closely linked to the chromosomal segments containing loci under selection for resistance. In the European Corn Borer (*O. nubilalis*), although no significant departure from Hardy–Weinberg equilibrium was initially identified (Bourguet et al. 2000), mating was found to take place at restricted spatial scales (within 50 m), effectively translating into an assortative mating rate of perhaps 5% (Dalecky et al. 2006; Bailey et al. 2007). Low premating movement is expected to increase the rate of assortative mating (*F *>* *0) between individuals originating from the same block of *Bt* crop and has been suggested in few moths (see Cuong and Cohen 2003; Qureshi et al. 2006). In addition, some *Bt*‐resistant pest strains evince slower larval development than *Bt*‐susceptible conspecifics, potentially leading to emergence asynchrony of resistant and susceptible genotypes (c.f., Gryspeirt and Grégoire 2012), which could increase assortative mating (in general) but also an elevated rate of mating with resistant siblings. We clearly need better information on pest ecology, and in particular, information on dispersal behavior, with respect to the various contexts within which transgenic crops are grown.

### Pace of resistance evolution and passage time

Both the pace of resistance evolution and the passage time can be described by simple combinations of model parameters. On the one hand, the expected rates of resistance evolution can be obtained by evaluating the (*V*_RR_, *U*_RR_, *ε*,* χ*, and *ω*) parameters when estimates of those parameters are attainable. On the other hand, the observed rates of evolution can be obtained by observing allele frequency changes under field conditions. That duality provides us with a simple framework for explicitly connecting empirical data and theory. While Δ*q*, as a measure of resistance evolution, is completely dependent on the allele frequency *q* at any particular point on the trajectory, the rate ξ* offers a standardized measure of the general pace of resistance evolution, even when precise estimates of (*ω*,* U*_RR_, *V*_RR_, *g*,* h* and *F*) are not available, provided that (*q* < 0.1) is below the ‘loss of containment’ threshold.

For the sake of illustration, we consider the case of *B. fusca* resistance in South Africa, for which no fitness cost has been observed (Kruger et al. 2014), and for which resistance seems complete and inherited as a dominant trait (Campagne et al. 2013). Moreover, the planted fraction of *Bt* maize averaged (*ω *< 0.30) from 1998 to 2004 (~14 generations) in the area where this resistance evolved (Tabashnik et al. 2009). Assuming that initial allele frequency was low (i.e., 0.0001 < *q*_0_ < 0.001), the expected pace of resistance would then be [*ω·ε·V*_RR_/(1 − *ω*)] ≈ [0.3*·*1*·*1/(1 − 0.3)] ≈ 0.43 (i.e., a passage time of ~11 to 16 generations, depending on *q*_0_), roughly compatible with an empiric estimate of ξ* ≈ 0.34 (i.e., ~14 generations), based on the rate of change in resistance frequency (Table 2).

## Implications for resistance management and monitoring

The main option for delaying resistance evolution is to manipulate the fraction of refuge crop, either its proportion (1 − *ω*), lowering selection pressure, or its spatial organization, reducing the impact of limited dispersal on *F *>* *0. In a context where resistance evolution is not expected to follow the trajectory of a ‘strictly recessive’ allele (i.e., when *ε *> 0), and where the estimation of some important parameters might not be achievable, robust resistance management might have to involve substantial refuge fractions. Vacher et al. (2003) suggested refuge fractions of ~25% to minimize pest density while efficiently delaying resistance evolution. Similarly, our results show that (1 − *ω*) < 0.20 are not likely to result in an effective expression of the fitness cost to increase passage time. Some strategies might even be needed to ensure that (Δ*y* ≤ 0). In this case, the minimum fraction of refuge preventing an increase in resistance allele frequency is expected to be (1 − Ω) > 30%. Unsprayed refuge requirements as low as (5%) of the total planted with *Bt* crops (Bates et al. 2005) do not appear to be sufficient with respect to the statements above (see also Vacher et al. 2003). By contrast, in the Southern states of the USA, where cotton is grown, the decision was taken to establish more generous refuge fractions, (1 − *ω*)* *≈ *ω *≈ 0.5, in areas where other *Bt* crops were deployed.

The notion of high dose toxin, in the context of *Bt* crops, relies on a purely empirical criterion, a dose that kills 99.99% of susceptible individuals in the field ‘to assure that 95% of heterozygotes would probably be killed’ (USEPA 1998, see also Gould 1998), which translates as (*ε *> *h *>* *0.05). In this respect, our model results provide rationale to expect rapid evolution of resistance (for *h *=* *0.05), typically requiring a high refuge fraction (1 − *ω* > 0.25) to achieve a passage time of *T* ^k^ ≈ 40 generations (with *q*_*0*_ = 0.001, *q*_*k*_ = 0.1, *F *=* *0, *V*_RR_ = 1, *U*_RR_ = 0.75, *g* = 0.1).

The model suggests that the definition of ‘high‐dose’ should depend explicitly on (the unknown) *q*_0_ [see equation (9b) and Appendix S1], since the variation in frequency of the resistance allele is inflated by a factor [1 + (*p*/*q*)*ε*] ≈ (1 + *ε*/*q*) whenever the system deviates from idealized HDR behavior. Assuming the parameters just above, if we set our ‘dosage requirement’ high enough to ensure that *ε *= (*F *+ *h* − *F·h*) < *q*_0_, which would reduce ($\tilde{\Lambda }\to 2\cdot \tilde{A}$) at most; we need a dose that kills a fraction *p*_0_ of RS heterozygotes. Our model (with the same parameters as above) shows that we can attain a passage time of *T* ^*k*^ ≈ 40 with a refuge fraction of only (1 − *ω*) = 5%, but only if we can assure that (*ε *< 0.007). Our findings suggest that, even with random mating, the current ‘high‐dose’ requirement is inadequate for low refuge fractions. The ‘dose’ or the refuge fraction (1 − *ω*) needs to be increased.

An efficient insect resistance management strategy must be based on robust assumptions that ensure sustained toxicity of *Bt* crops under a variety of circumstances. Notably, insect survival on transgenic crops expressing at least two *Bt* toxins appeared to be higher than previously anticipated (Carrière et al. 2015). In this context, both, breeding programs and modeling studies may benefit from explicitly integrating other deviations from idealized situations in order to minimize the gap between theoretical expectations and empirical trends observed in the field. Better predictive models of resistance evolution may be a key for both designing sustainable strategies and anticipating eventual failures.

## Competing interests

The authors declare no competing interests.

## Data accessibility

This study did not involve unpublished empirical data.

## Supporting information


**Appendix S1.** Model derivations.

**Appendix S2.** Passage time approximations.
