# Supplementary material for: Impact of violated high‐dose refuge assumptions on evolution of Bt resistance
Source: Evol Appl. 2016 Feb 27;9(4):596–607. doi: 10.1111/eva.12355 (PMC4831461; doi:10.1111/eva.12355)
Supplement: Supplementary file 1 — Appendix S1. Model derivations. [file EVA-9-596-s001.pdf]

## Appendix A: Model derivations

Here we derive four different models, corresponding to cases where $F$ = 0 or > 0 and/or where $h$ = 0 or > 0, but holding homozygote fitness values to either 0 or 1 (see Tables below).

| | |
|---|---|
| **Case 1: ($F$ = 0 = $h$)** | **Case 2: ($F$ > 0 = $h$)** |
| **Case 3: ($F$ = 0 < $h$)** | **Case 4: ($F$ > 0 < $h$)** |

### Case 1: ($F$ = 0 = $h$)

| **Model Parameters** | **SS** | **RS** | **RR** | **Planting Fraction** |
|---|---|---|---|---|
| Frequencies | $p^2$ | $2pq$ | $q^2$ | |
| *Bt*-Fitness | $V_{SS} = 0$ | $V_{RS} = 0$ | $V_{RR} = 1$ | ω |
| Refuge Fitness | $U_{SS} = 1$ | $U_{RS} = 1$ | $U_{RR} = 1$ | (1 – ω) |

**Weighted Average Allelic Fitness Values**

$$\tilde{V}_S = p\left[p\cdot V_{SS} + q\cdot V_{RS}\right] = 0 \qquad \tilde{V}_R = q\cdot\left[q\cdot V_{RR} + p\cdot V_{RS}\right] = q^2$$

$$\tilde{U}_S = p\cdot\left[p\cdot U_{SS} + q\cdot U_{RS}\right] = p \qquad \tilde{U}_R = q\cdot\left[q\cdot U_{RR} + p\cdot U_{RS}\right] = q$$

$$\tilde{W}_S = \omega\cdot\tilde{V}_S + (1-\omega)\cdot\tilde{U}_S = (1-\omega)\cdot p \qquad \tilde{W}_R = \omega\cdot\tilde{V}_R + (1-\omega)\cdot\tilde{U}_R = \omega\cdot q^2 - (1-\omega)\cdot q$$

Defining $\overline{W} = (\tilde{W}_S + \tilde{W}_R)$, we start with $p' = \tilde{W}_S / \overline{W}$ and $q' = \tilde{W}_R / \overline{W}$. Using the equivalent form $y$ = ( $q$ / $p$ ), we define $y' = \left(p'/q'\right) = \tilde{W}_R/\tilde{W}_S$, which leads to:

$$y' = \left[\frac{(1-\omega)+q\cdot\omega}{(1-\omega)}\cdot\frac{q}{p}\right] = \frac{(1-\omega)+q\cdot w}{(1-\omega)}\cdot y = \left[\frac{q\cdot\omega}{(1-\omega)}+1\right] = \left[\tilde{A}+1\right]\cdot y \qquad \text{[A1]}$$

and thus

$$\Delta y = \left(y' - y\right) = \tilde{A} = \left[\frac{q\cdot\omega}{(1-\omega)}\right] \quad . \qquad \text{[A2]}$$

The pace of resistance evolution *increases* with the fraction of acreage planted to the *Bt*-crop.

## Appendix A: Model derivations

### Case 2: ($F > 0 = h$)

| Model Parameters | SS | RS | RR | Planting Fraction |
|---|---|---|---|---|
| Frequencies | $p^2 + pqF$ | $2pq(1 - F)$ | $q^2 + pqF$ | |
| *Bt*-Fitness | $V_{SS} = 0$ | $V_{RS} = 0$ | $V_{RR} = 1$ | $\omega$ |
| Refuge Fitness | $U_{SS} = 1$ | $U_{RS} = 1$ | $U_{RR} = 1$ | $(1 - \omega)$ |

**Weighted Average Allelic Fitness Values**

$$\tilde{V}_S = 0 \qquad \tilde{V}_R = q \cdot (q + pF)$$

$$\tilde{U}_S = p \cdot \left[ (p + qF) + q \cdot (1 - F) \right] = p \qquad \tilde{U}_R = q \cdot \left[ (q + pF) + p(1 - F) \right] = q$$

$$\tilde{W}_S = (1 - \omega) \cdot p \qquad \tilde{W}_R = q \cdot \left[ (1 - \omega) + (q + pF) \cdot \omega \right]$$

That leads us instead to:

$$y' = \left[ \frac{q}{p} \cdot \frac{(1-\omega) + (q + pF) \cdot q\omega}{(1-\omega)} \right] = \left[ \frac{q^2 \cdot \omega}{(1-\omega)} \cdot \left( 1 + \frac{q}{p} \cdot F \right) + 1 \right] \cdot y = \left[ \tilde{B} + 1 \right] \cdot y \qquad \text{[A3]}$$

It is then possible to define $\Delta y = (y' - y)$, and express it as an explicit function of $\tilde{B}$:

$$\Delta y = \tilde{B}, \quad \text{where} \quad \tilde{B} = \left[ 1 + \left( \frac{p}{q} \right) \cdot F \right] \cdot \tilde{A}, \qquad \text{[A4]}$$

which also yields:

$$\frac{\Delta y_{F>0}}{\Delta y_{F=0}} = \frac{\tilde{B}}{\tilde{A}} = \left[ 1 + \left( \frac{p}{q} \right) \cdot F \right] >> 1 \quad . \qquad \text{[A5]}$$

The frequency of R mutant is small at the outset, so ($q_0 << p_0$), and $y_0$ is very large. Even small values of ($F > 0$) exhibit profoundly elevated rates of resistance evolution, relative to **Case I**.

## Appendix A: Model derivations

### Case 3: ($F = 0 < h$)

| Model Parameters | SS | RS | RR | Planting Fraction |
|---|---|---|---|---|
| Frequencies | $p^2$ | $2pq$ | $q^2$ | |
| *Bt*-Fitness | $V_{SS} = 0$ | $V_{RS} = h$ | 1 | ω |
| Refuge Fitness | $U_{SS} = 1$ | $U_{RS} = 1$ | $U_{RR} = 1$ | (1 – ω) |

**Weighted Average Allelic Fitness Values**

$$\tilde{V}_S = p \cdot [p \cdot V_{SS} + q \cdot V_{RS}] = p \cdot q \cdot h \qquad \tilde{V}_R = q \cdot [q \cdot V_{RR} + p \cdot V_{RS}] = q \cdot [q + p \cdot h]$$

$$\tilde{U}_S = p \cdot [p + q \cdot U_{RS}] = p \qquad \tilde{U}_R = q \cdot [q \cdot U_{RR} + p \cdot U_{RS}] = q$$

$$\tilde{W}_S = p \cdot [(1-\omega) + q \cdot h \cdot \omega] \qquad \tilde{W}_R = q \cdot [(1-\omega) + (q + p \cdot h) \cdot \omega]$$

The model leads to:

$$y' = \left( \frac{\tilde{W}_R}{\tilde{W}_S} \right) = \left[ \frac{(1-\omega) + (q + ph) \cdot \omega}{(1-\omega) + qh\omega} \right] \cdot y \ . \qquad \text{[A6]}$$

Factoring by $q$ and $(1 - \omega)$, and simplifying, we obtain:

$$y' = \frac{\left[ 1 + \left( 1 + \frac{p}{q} \cdot h \right) \cdot \tilde{A} \right]}{1 + h \cdot \tilde{A}} \cdot y \approx \frac{\left[ 1 + \tilde{C} \right]}{1 + h \cdot \tilde{A}} \cdot y \ , \qquad \text{[A7]}$$

but for $q$ and $h$ both small, $(1 + h \cdot \tilde{A}) \approx 1$, which leads us to:

$$\Delta y \approx \tilde{C} \ \text{, where } \tilde{C} = \left[ 1 + \frac{p}{q} \cdot h \right] \cdot \tilde{A} \ , \qquad \text{[A8]}$$

and the rate of resistance evolution is again vastly elevated, relative to the HDR model, since

$$\frac{\Delta y_{h>0}}{\Delta y_{h=0}} = \frac{\tilde{C}}{\tilde{A}} \approx \left[ 1 + \frac{p}{q} \cdot h \right] >> 1 \ . \qquad \text{[A9]}$$

**Case 4: ($F$ > 0 < $h$)**

| Model Parameters | SS | RS | RR | Planting Fraction |
|---|---|---|---|---|
| Frequencies | $p^2 + pqF$ | $2pq(1 - F)$ | $q^2 + pqF$ | |
| *Bt*-Fitness | $V_{SS} = 0$ | $V_{RS} = h$ | $V_{RR} = 1$ | ω |
| Refuge Fitness | $U_{SS} = 1$ | $U_{RS} = 1$ | $U_{RR} = 1$ | (1 – ω) |

**Weighted Average Allelic Fitness Values**

$$\tilde{V}_S = p \cdot \left[ q \cdot (1-F) \cdot h \right] \qquad \tilde{V}_R = q\left[ q + p\left(F + h - Fh\right)\right]$$

$$\tilde{U}_S = p \cdot \left[ (p + qF) \cdot U_{SS} + q \cdot (1-F) \cdot U_{RS} \right] = p \qquad \tilde{U}_R = q \cdot \left[ (q + pF) \cdot U_{RR} + p(1-F) \cdot U_{RS} \right] = q$$

$$\tilde{W}_S = p \cdot \left[(1-\omega) + (1-F) \cdot hq\omega\right] \qquad \tilde{W}_R = q \cdot \left[(1-\omega) + (q + pF) \cdot \omega + (1-F) \cdot hp\omega\right]$$

For this most general case, we discover again that $y'$ may be written as a function of Ã:

$$y' = \frac{\left[1 + \left[1 + \frac{p}{q} \cdot \left(F + h - F \cdot h\right)\right] \cdot \tilde{A}\right]}{1 + (1-F) \cdot h \cdot \tilde{A}} \cdot y \qquad \text{[A10]}$$

and for $q$ and $h$ both small, the denominator → 1, all of which leads to

$$\Delta q \approx \tilde{D} \text{ , where } \quad \tilde{D} = \left[1 + \frac{p}{q} \cdot \left(F + h - F \cdot h\right)\right] \cdot \tilde{A} \text{ ,} \qquad \text{[A11]}$$

from which we can also derive

$$\frac{\Delta y_{F>0<h}}{\Delta y_{F=0=h}} = \frac{\tilde{D}}{\tilde{A}} \approx \left[1 + \left(\frac{p}{q}\right) \cdot \left(F + h - F \cdot h\right)\right] >> 1 \text{ ,} \qquad \text{[A12]}$$

almost a doubly elevated rate of resistance evolution.
