# Supplementary material for: Impact of violated high‐dose refuge assumptions on evolution of Bt resistance
Source: Evol Appl. 2016 Feb 27;9(4):596–607. doi: 10.1111/eva.12355 (PMC4831461; doi:10.1111/eva.12355)
Supplement: Supplementary file 2 — Appendix S2. Passage time approximations. [file EVA-9-596-s002.pdf]

Appendix B: Passage Time approximations

HDR Model (*F* = 0 = *h*):

We can approximate the difference equation for the simple HDR (ε = 0) model with a 1st order ordinary differential equation. Thus, we can write

$$\Delta y \approx \frac{\delta y}{\delta t} = \tilde{\mathrm{A}} \cdot y$$

where $\tilde{\mathrm{A}} = \left(\frac{q \cdot \omega}{1-\omega}\right) = \left(\frac{\omega}{1-\omega}\right) \cdot \frac{y}{1+y}$, which yields the following solution:

$$t = \left(\frac{1-\omega}{\omega}\right) \cdot \left[-\frac{1}{y_t} + \ln(y_t) + c\right] \qquad \text{[B1]}$$

where c is a constant of integration, determined by initial conditions, and t is expressed in continuous generations. Note that, as long as $y_t > 0$, Eq. [B1] can be re-written as:

$$\left(\frac{p_t}{q_t}\right) \cdot \exp\left\{-\frac{p_t}{q_t}\right\} = \exp\left\{-\left(\frac{\omega}{1-\omega} \cdot t + c\right)\right\} \qquad \text{[B2]}$$

which is equivalent to (see also Fig. B1):

$$y_t = \frac{1}{\mathbf{W}\left(e^{-\left(\frac{\omega}{1-\omega} \cdot t + c\right)}\right)} \qquad \text{[B3]}$$

where **W** is Lambert's W function which reduces $\mathbf{W}\left(y_t \cdot e^{y_t}\right)$ to y_t, and where c is the constant of integration. Based on (Figure B.1), which plots the temporal trajectory of $q_t = y_t / (1 + y_t)$, we define the "passage time" (T_0^k, in generations) required to go from (q_0 to q_k) for the classic HDR (ε = 0) model as:

$$T_0^k = t_k - t_0 = \left(\frac{1-\omega}{\omega}\right) \cdot \left[\frac{1}{y_0} - \frac{1}{y_k} + \ln\left(\frac{y_k}{y_0}\right)\right] \quad . \qquad \text{[B4]}$$

[Figure B.1 - about here]

Appendix B: Passage Time approximations

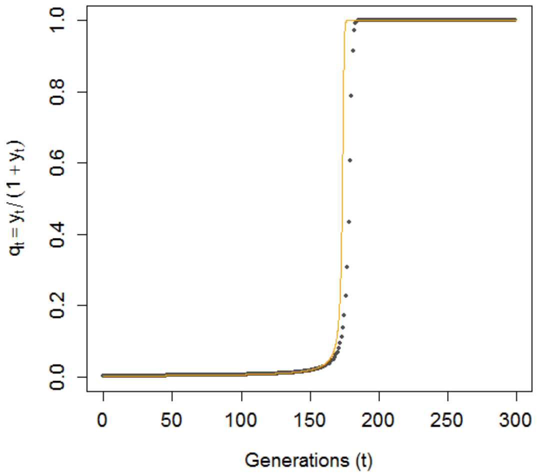

Fig. B1: Frequency of a recessive resistance allele as a function of time. Dots represent the trajectory obtained using iterations (see Eq. [3a,b]), continuous line represents the continuous-time approximation (Eq. [B3]). The parameters where set as follow: ω = 0.7, q_0 = 0.0025.

Generalized Model

The increase of resistance allele frequency in a single generation can be calculated based on Δy (see Text Table 1 for the labels of the different parameters):

$$\Delta y = (y' - y) = \left[\frac{\tilde{W}_R}{\tilde{W}_S} - y\right] = \left[\frac{\omega \cdot \tilde{V}_R + (1-\omega)\cdot \tilde{U}_R}{\omega \cdot \tilde{V}_S + (1-\omega)\cdot \tilde{U}_S} - 1\right] \cdot y = \tilde{\Lambda} \cdot y \qquad \text{[B5]}$$

which is compatible with all the special cases, since $\tilde{\Lambda}$ translates into $\tilde{A}$, $\tilde{B}$, $\tilde{C}$ and $\tilde{D}$ for text Eqq. [5b], [6a], [7a] and [8a], respectively. To solve the differential equation based on the difference equation [B5], $\tilde{\Lambda}$ may be easily re-written as a ratio of two linear functions of *y*,

$$\tilde{\Lambda} = \frac{(\beta_1 \cdot y) + \gamma_1}{(\beta_2 \cdot y) + \gamma_2} - 1 \qquad \text{[B6]}$$

where

Appendix B: Passage Time approximations

$$\begin{aligned}
\beta_1 &= \omega \cdot \mathrm{V_{RR}} + (1-\omega) \cdot \mathrm{U_{RR}} \\
\gamma_1 &= \omega \cdot \left[\mathrm{F} \cdot \mathrm{V_{RR}} + (1-\mathrm{F}) \cdot \mathrm{V_{RS}}\right] + (1-\omega) \cdot \left[\mathrm{F} \cdot \mathrm{U_{RR}} + (1-\mathrm{F}) \cdot \mathrm{U_{RS}}\right] \\
\beta_2 &= \omega \cdot \left[\mathrm{F} \cdot \mathrm{V_{SS}} + (1-\mathrm{F}) \cdot \mathrm{V_{RS}}\right] + (1-\omega) \cdot \left[\mathrm{F} \cdot \mathrm{U_{SS}} + (1-\mathrm{F}) \cdot \mathrm{U_{RS}}\right] \\
\gamma_2 &= \omega \cdot \mathrm{V_{SS}} + (1-\omega) \cdot \mathrm{U_{SS}}
\end{aligned} \qquad [B7]$$

leading to:

$$\frac{\delta y}{\delta x} = \tilde{\Lambda} \cdot y = \frac{(\beta_1 - \beta_2) \cdot y + (\gamma_1 - \gamma_2)}{\beta_2 \cdot y + \gamma_2} \cdot y \ , \qquad [B8]$$

Solution of the differential equation [B8] yields an expression for passage time:

$$T_\Lambda^k \approx \frac{\gamma_2}{\gamma_1 - \gamma_2} \cdot \ln\left(\frac{y_k}{y_0}\right) + \frac{(\beta_1 \cdot \gamma_2 - \beta_2 \cdot \gamma_1)}{(\beta_2 - \beta_1)(\gamma_2 - \gamma_1)} \cdot \ln\left(\frac{(\beta_2 - \beta_1) \cdot y_k + (\gamma_2 - \gamma_1)}{(\beta_2 - \beta_1) \cdot y_0 + (\gamma_2 - \gamma_1)}\right) \qquad [B9]$$

For y_0 small and $y_k < 0.1$, however, the expression can be simplified, since the 2nd order terms may be neglected in Text Eq.[11b]. Retaining only the first term of text Eq. [13], we achieve a further approximation of T_Λ^k for the case where $\mathrm{V_{SS}} = 0$ and $\mathrm{U_{SS}} = 1$ (the reference fitness values):

$$T_\Lambda^k \approx \frac{\gamma_2}{\gamma_1 - \gamma_2} \cdot \ln\left(\frac{y_k}{y_0}\right) = \frac{1-\omega}{\omega \cdot \varepsilon \cdot \mathrm{V_{RR}} - (1-\omega) \cdot \chi \cdot (1 - \mathrm{U_{RR}})} \cdot \ln\left(\frac{y_k}{y_0}\right) \ , \qquad [B10]$$

where $\varepsilon = (F + h - F \cdot h)$, as before, and where $\chi = (F + g - F \cdot g)$.

Performing a simple simulation, we show that the approximation in Eq [B10] is a suitable lower-bound estimate of the full expression Eq [B9] when the number of generations considered is ($T_\Lambda^k < 100$), as shown in (Figure B2 - see below).

[Figure B2 – about here]

Appendix B: Passage Time approximations

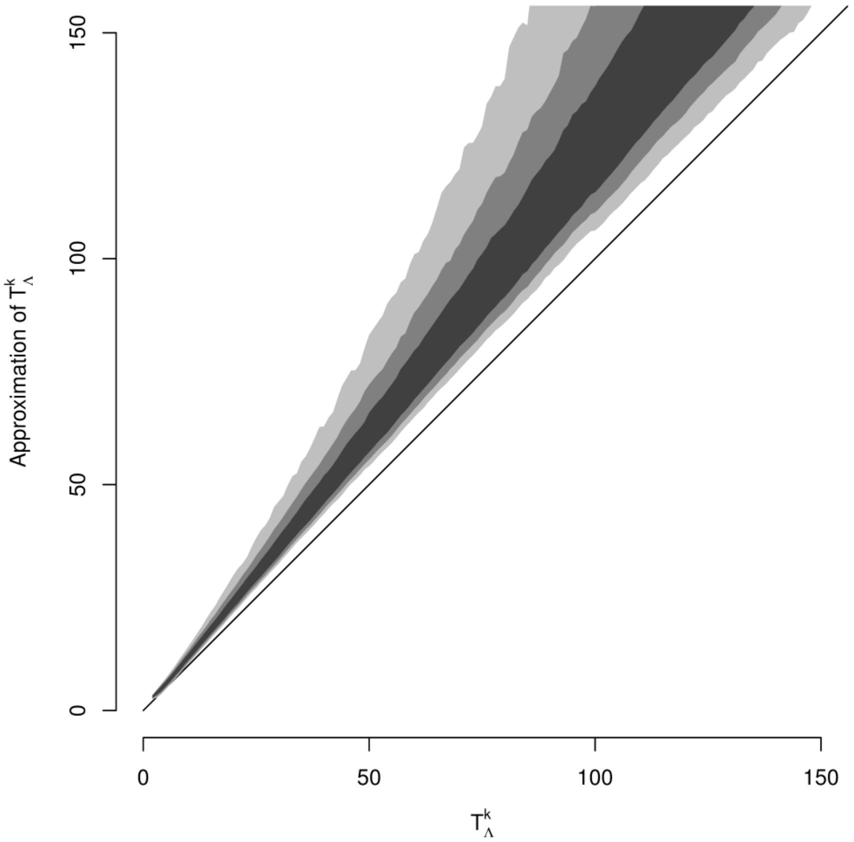

Figure B2: Approximation of passage time Eq. [B10] as compared to the numerical values of the full expression [B9]. 100,000 simulations were performed by randomly sampling values of the model parameters from uniform distributions: ($0.5 < \omega < 0.95$); ($0.5 < V_{RR} < 1$); ($0.5 < U_{RR} < 1$); ($0.0001 < q_0 < 0.005$); ($0 < g < 0.25$); ($0 < h < 0.1$); ($0 < F < 0.1$), while $U_{SS} = 1$ and $V_{SS} = 0$. Calculations were performed for both the approximation and the full expression, on each of the 100,000 parameters set. Grey envelopes represent symmetric simulation intervals: light grey envelope contains 90% of simulated T_Λ^k approximations; middle grey, 75%; dark grey, 50%.
